# G Protein Subunit Dissociation and Translocation Regulate Cellular Response to Receptor Stimulation

**DOI:** 10.1371/journal.pone.0007797

**Published:** 2009-11-11

**Authors:** Mariangela Chisari, Deepak Kumar Saini, Joon-Ho Cho, Vani Kalyanaraman, N. Gautam

**Affiliations:** 1 Department of Anesthesiology, Washington University School of Medicine, St. Louis, Missouri, United States of America; 2 Department of Genetics, Washington University School of Medicine, St. Louis, Missouri, United States of America; University of California, Berkeley, United States of America

## Abstract

We examined the role of G proteins in modulating the response of living cells to receptor activation. The response of an effector, phospholipase C-β to M3 muscarinic receptor activation was measured using sensors that detect the generation of inositol triphosphate or diacylglycerol. The recently discovered translocation of Gβγ from plasma membrane to endomembranes on receptor activation attenuated this response. A FRET based G protein sensor suggested that in contrast to translocating Gβγ, non-translocating Gβγ subunits do not dissociate from the αq subunit on receptor activation leading to prolonged retention of the heterotrimer state and an accentuated response. M3 receptors with tethered αq induced differential responses to receptor activation in cells with or without an endogenous translocation capable γ subunit. G protein heterotrimer dissociation and βγ translocation are thus unanticipated modulators of the intensity of a cell's response to an extracellular signal.

## Introduction

G proteins are the major modulators of cellular responses to external signals in mammalian cells [1], [2]. There is limited information on the role that G proteins play in directly regulating the sensitivity of a cell's response to an external stimulus in living cells. Studies in intact yeast cells [3] and in a mammalian cell line [4] have attempted to address the quantitative relationship between G protein activation and activity downstream. The kinetics of the rod photoreceptor G protein, Gt, mediated phototransduction activity has also been examined in highly specialized rod photoreceptor cells which are amenable to such studies [5]. Overall however, little is known about such processes with regard to the large families of G protein subunits that are expressed widely in all mammalian cell types. Here we have used imaging methods to examine whether mechanisms at the level of the G protein subunits control the intensity of the response to receptor activation in intact living cells.

Recently we demonstrated that on receptor activation, a family of G protein βγ complexes translocates from the plasma membrane to endomembranes and reverse translocates when a receptor is deactivated [6], [7]. The translocation reduces the concentration of Gβγ on the plasma membrane rapidly (t½ ∼10 s) [6], [7]. We expected this reduced concentration of Gβγ on the plasma membrane would attenuate the response of an effector, phospholipase β (PLCβ) to the activation of a receptor coupled to the G protein, Gq. We tested this hypothesis using live cell imaging experiments using the PLCβ mediated signaling pathway.

M3 muscarinic receptor activation of the Gαq activates phospholipases C β isozymes (PLCs), leading to phosphatidylinositol (4,5)-bisphosphate (PIP2) hydrolysis on the plasma membrane resulting in the production of inositol triphosphate (IP3) and diacylglycerol (DAG) [8]. PIP2 breakdown is detected quantitatively in living cells by imaging the translocation of a pleckstrin homology (PH) domain of PLCδ tagged with a fluorescent protein, mCherry (mCh) [9] or a DAG binding domain of protein kinase C β (DBD) tagged with yellow fluorescent protein (YFP) [10], [11], [12]. When M3 receptors are stimulated, PH-mCh translocates from the plasma membrane to the cytosol because it binds with higher affinity to IP3 in the cytosol compared to PIP2 in the plasma membrane [13]. YFP-DBD translocates in the opposite direction from the cytosol to the plasma membrane because it binds to DAG [11]. Using these sensors we examined the impact of activating G proteins containing translocating and non-translocating βγ complexes on downstream PLC β effector response. We then used a fluorescence resonance energy transfer (FRET) based G protein sensor similar to a previous sensor [14] containing different γ subunits to identify the mechanistic basis of response differences. Finally, to evaluate the relevance of our observation at physiological concentrations of Gβγ, we used an αq subunit tethered to the M3 receptor to evaluate the effect of endogenous translocating and non – translocating Gγ subunits.

## Results

### Functionality of fluorescently tagged fusion proteins

**Figure 1:**
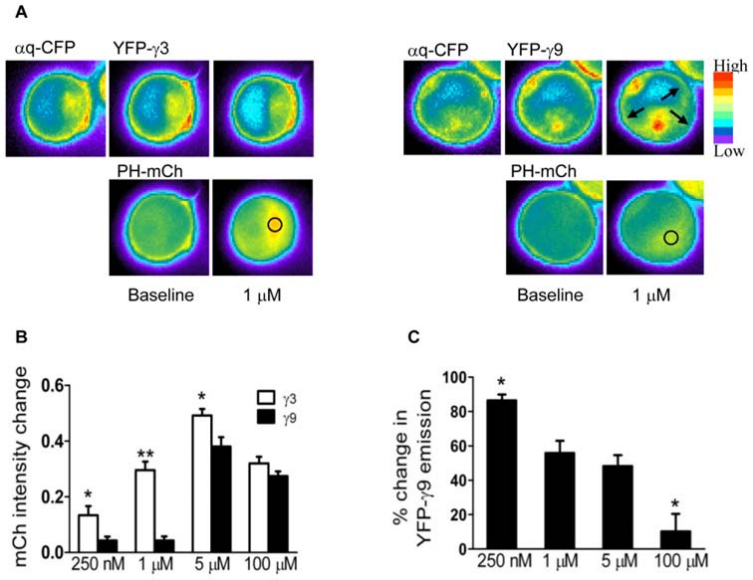
Responses of cells expressing various γ subunits to different concentrations of agonist. *A.* M3-CHO cells expressing αq-CFP, YFP-γ (as indicated) and PH-mCh were imaged as mentioned in Methods and Text S1. Representative images of cells exposed to 1 µM carbachol (agonist) are shown. The decrease in YFP-γ3 intensity observed is due to bleaching during acquisition. *B.* Translocation of PH-mCh at specific agonist concentrations. Bars show changes for PH-mCh translocation in the presence of various γ subunits. No. of γ3 cells examined at 250 nM–20; at 1 µM–11; at 5 µM–17 and at 100 µM–17. No. of γ9 cells examined at 250 nM–23; at 1 µM–11; at 5 µM–17 and at 100 µM–14. Cells with comparable expression of PH-mCh were imaged. Regions in the cytosol were selected for analysis (as indicated by circles). Values were calculated as ratio of M-B/B, where M is the maximal intensity at a particular concentration and B, the intensity before exposure to that concentration of agonist. Results are the means ± SEM. *p<0.01, **p<0.001. *C.* Translocation of YFP-γ9 subunits at specific agonist concentrations. Bar diagrams show changes in YFP-γ9 emission on the plasma membrane (N = 4). Regions on the plasma membrane were selected for analysis (as indicated by arrows in image, Fig. 1A, right panel). Values are shown as percentile of means ± SEM. *p<0.01, (response to 250 nM and 100 µM to others).

Fluorescently tagged G protein γ subunits used in this study have been previously described [6], [7] and have been shown to be functionally active [14], [15], [16], [17]. We also confirmed that the αq-CFP fusion protein was functional by examining PH-mCh translocation in M3-CHO cells which were transfected with αq-CFP and PH-mCh. PH-mCh translocates from the plasma membrane to the cytosol on PLC β activation and the magnitude of PH-mCh translocation is indicative of extent of PIP2 breakdown on the PM. In cells containing αq-CFP, the PH – domain translocation was significantly higher after receptor activation compared to untransfected cells (emission intensity in the cytosol was ∼1.6 fold higher (data not shown)) indicating that the introduced αq-CFP was active. We further confirmed functionality of αq-CFP by evaluating its ability to support translocation of the βγ complex. Endogenous αq does not support visually detectable βγ9 translocation (data not shown). This absence of background translocation of βγ9 induced by endogenous αq allowed us to examine whether the tagged αq-CFP was functional. We introduced αq-CFP into M3-CHO cells along with YFP tagged βγ9. On activation of M3 receptor in these cells, βγ9 translocated from the plasma membrane to the intracellular membranes (Fig. 1A, right panel). These results showed that αq-CFP fusion protein is functionally active.

### PH domain translocation is reduced in the presence of a translocating G protein γ subunit

**Figure 2:**
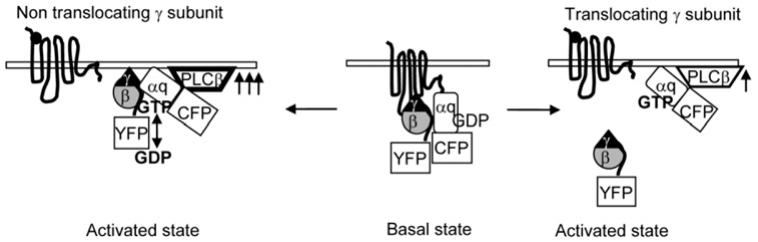
Model for the mechanistic basis of differential sensitivity of cells containing different γ subunits. On activation, G proteins progress through two different paths: (i) when they contain a non-translocating γ subunit (left) or (ii) when they contain a translocating γ subunit (right). Left: the activated α and βγ remain on the plasma membrane. Since receptor activation requires the heterotrimer, this results in continual activation of Gαq reflected in a high level of PLCβ activation (multiple arrows). Right, the translocation of βγ away from the plasma membrane reduces the heterotrimer concentration and subsequent PLCβ stimulation. Although, we have been unable to obtain evidence for βγ activation of PLCβ in these cells (Text S2), this model is consistent with the results even if βγ activates PLCβ because βγ concentration available for PLCβ activation is lowered by translocation.

To test the hypothesis that γ subunit translocation modifies the sensitivity of a cell's response to an agonist, PIP2 breakdown was measured at various concentrations of agonist. Gq containing different γ subunits tagged with fluorescent proteins were cotransfected into cells stably expressing M3 muscarinic receptors (M3-CHO) [14] along with the PH-mCh. Individual cells with similar emission levels of CFP and YFP were chosen for analysis. Cells were exposed independently to concentrations of agonist ranging from 250 nM to 100 µM. Buffer was introduced after agonist activation to deactivate the receptor. Emission intensities from the cytosol for PH-mCh and from intracellular membranes for YFP-γ subunits were measured to quantify the extent of translocation of these proteins from the plasma membrane where they were initially localized (Fig. 1A). For measuring PH-mCh changes we selected regions of approximately equal size in the cytosol. We ensured that the changes within selected regions were reflected overall in the cell cytosol and not restricted to the region selected (Fig. 1A). Images (Fig. 1A) and bar diagram (Fig. 1B) show the extent of PH-mCh translocation in the presence of the γ3 or γ9 subunits when cells were exposed to individual agonist concentrations (as indicated). Cells expressing γ3 at subsaturating agonist concentrations of 250 nM and 1 µM showed a response that was significantly higher compared to cells expressing γ9 (Fig. 1B). This difference is seen even at 5 µM concentration (Fig. 1B). The PIP2 breakdown stimulated by the M3 receptor begins to saturate above 5 µM (Fig. 1B). Fig. 1C shows the magnitude of YFP-γ9 translocation determined by measuring the fluorescence intensity in the plasma membrane before and after agonist addition. The YFP emission intensity on the plasma membrane was calculated as the average of selected regions as shown in the example in Fig. 1A. The magnitude of βγ translocation is directly correlated with increasing agonist concentration. These results together support the model that the magnitude of cellular response is decreased by the removal of the βγ complex from the plasma membrane on receptor stimulation (Fig. 2). Conversely, cells expressing non-translocating γ subunits show higher sensitivity to an agonist (Fig. 2).

### The cumulative response of cells expressing γ3 and γ9 to sequential addition of increasing concentrations of agonist are the same

**Figure 3:**
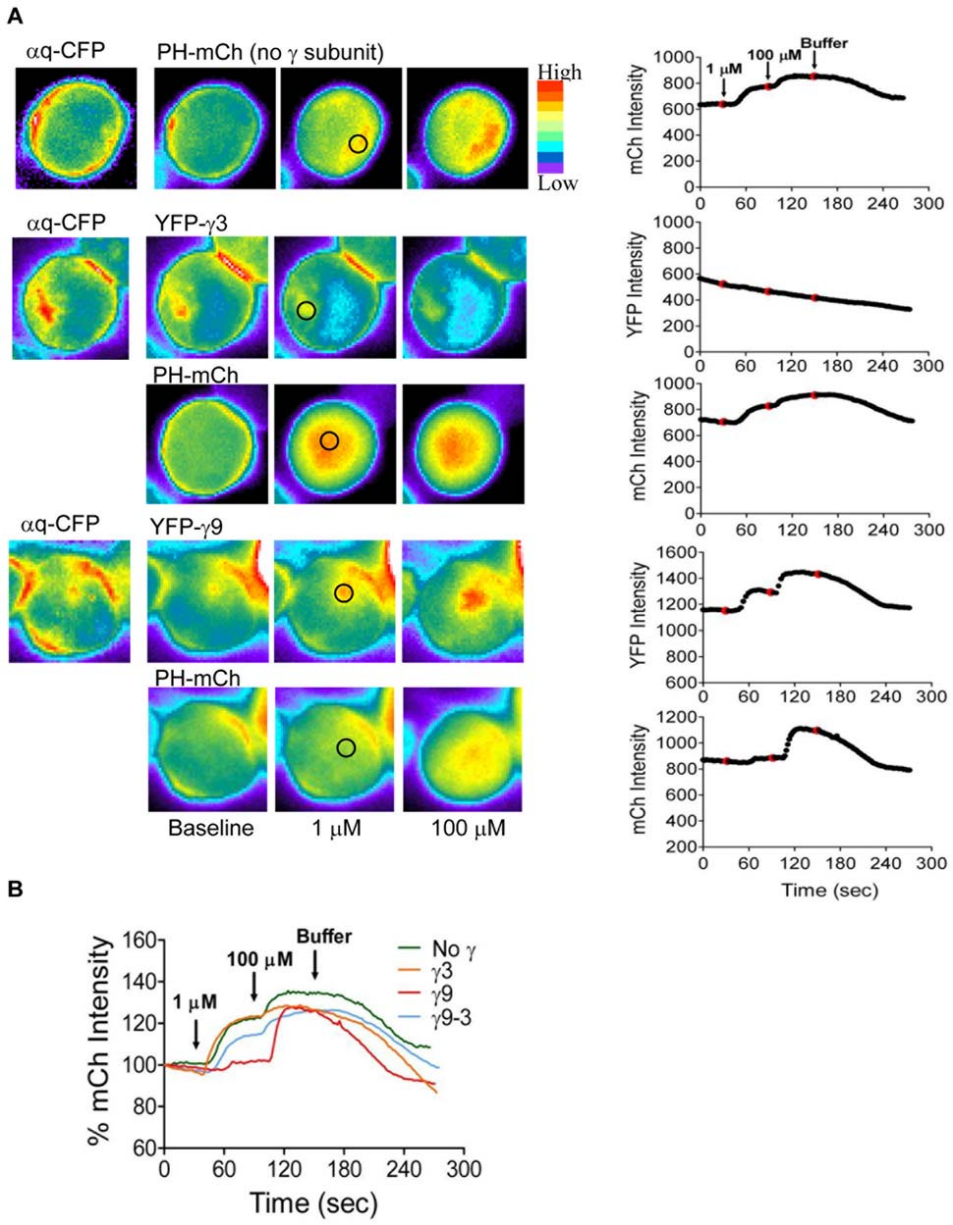
Responses of cells expressing different γ subunits to sequential exposure of increasing concentrations of agonist. *A.* M3-CHO cells expressing αq-CFP, YFP-γ (as indicated) and PH-mCh were imaged as mentioned in Methods and Text S1. Representative images of cells exposed sequentially to 1 µM and 100 µM carbachol (agonist) are shown. Changes were measured in selected regions (black circles) in cytosol (for mCh) and endomembranes (for YFP). In the corresponding plots on the right, arrows in the top plot and red dots in all plots, indicate time points at which agonist and buffer were introduced. *B.* Plots of PH-mCh emission intensity for cells expressing different γ subunit types (as indicated). Basal level of mCh intensity was treated as 100%.

We then measured the cumulative response of these cells to the sequential exposure of cells to 1 µM and 100 µM. While no detectable translocation of the γ3 subunit was observed as reported previously[7] (Fig. 3A), γ9 translocated in response to the initial stimulus with 1 µM and then to the subsequent addition of 100 µM agonist (Fig. 3A). In cells expressing YFP-γ3 or endogenous γ (no introduced γ subunit), the amount of PH-mCh translocation at 1 µM was very strong compared to those cells expressing YFP-γ9. With the sequential addition of higher concentration of carbachol (100 µM), the additional PH-mCh translocation in the presence of γ3 or endogenous γ showed a small increase. In contrast, the response to 100 µM agonist was significantly stronger in cells co-expressing γ9 (Fig. 3A). In Fig. 3B representative plots of the time dependent responses of individual cells to the sequential addition of agonist are compared for cells expressing different γ subunits. This comparison shows that the cumulative response to the sequential addition of 1 µM and 100 µM agonist is similar in all cells. Overall these results show that the differential response of cells expressing different γ subunits to low agonist concentrations is not due to inhibition of PIP2 breakdown.

### Cells expressing a chimeric γ9-3 subunit which is incapable of translocation shows heightened receptor sensitivity compared to γ9

**Figure 4:**
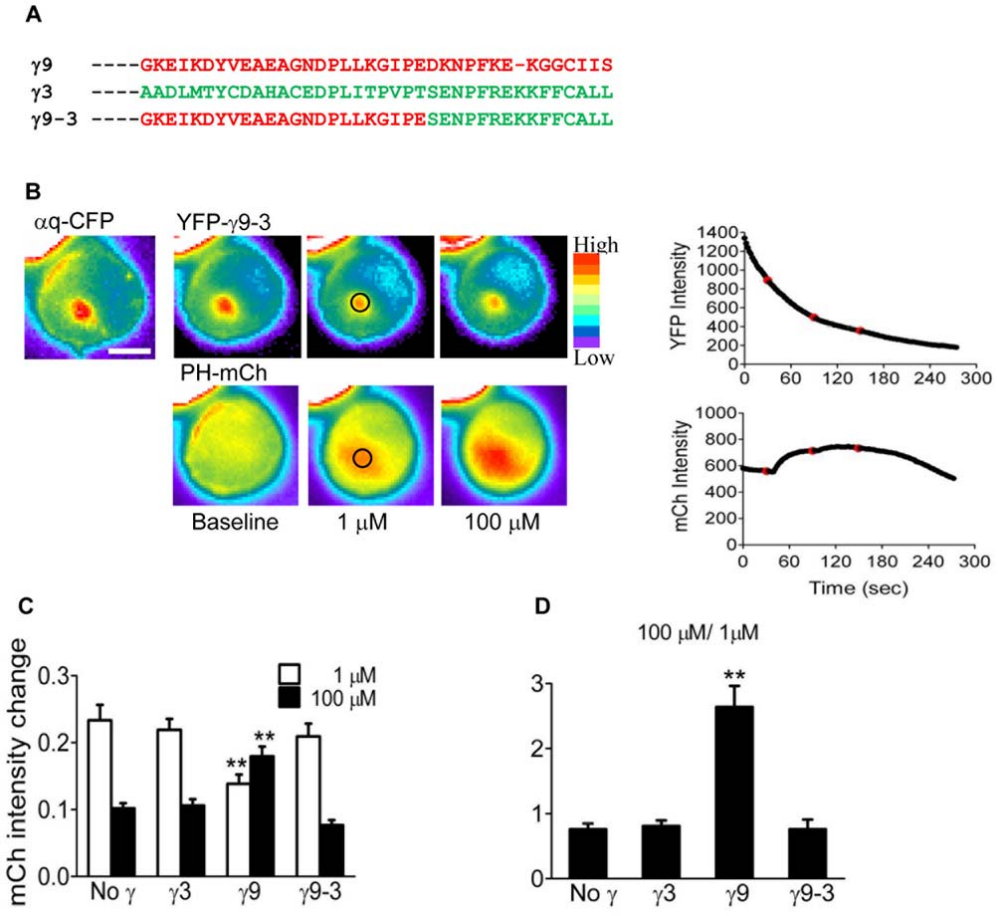
Response of a γ9-3 chimera. *A.* C terminal sequences of relevant γ subtypes. *B.* Representative images and plots of γ9-3 chimera and PH-mCh translocation. Experiments and analysis were performed as described in Fig. 3A . *C.* Bar diagrams of the extent of PH-mCh translocation at different concentrations of agonist in cells expressing indicated γ subunits. Values were calculated as in Fig. 1B *D.* Ratio of the 100 µM over 1 µM response. Results are represented as mean ± SEM (N =  no γ-51, γ3-52, γ9-54, γ9-3 -40). **p<0.001 (γ9 versus others at the same concentration).

We then examined whether the differential impact of γ subunits on PIP2 breakdown is due to unidentified differential roles of the γ3 and γ9 subunit types or due to the translocation of the βγ9 complex away from the plasma membrane to internal membranes. To confirm the role of βγ translocation we constructed a chimeric γ9 subunit in which the C terminal 15 residues of the translocation proficient γ9 were replaced with the corresponding sequence of γ3 which does not translocate (γ9-3, Fig. 4A). We have previously shown that the C terminal domain of a γ subunit controls its translocation capability [7], [18]. The chimeric molecule, γ9-3, did not translocate in response to receptor activation thus acquiring the properties of γ3 while retaining most of the residues of γ9 upstream of the C terminal 15 residue domain (Fig. 4B). We analyzed the receptor stimulated translocation of the PH domain in the presence of γ9-3 in selected cells as described above. Responses to both 1 µM and 100 µM agonist, added sequentially, were measured. PH-mCh translocation in the presence of the γ9-3 subunit (Fig. 4B) was similar to that observed in the presence of γ3 or endogenous γ subunits in CHO cells (Fig. 3A and B). This result clearly shows that receptor stimulated downstream activity is controlled by the translocation of the βγ complex away from the plasma membrane and is not an unusual property of a particular γ subunit type.

The results of the experiments using sequential addition of agonists was examined further to compare the effects of the γ subunit constitution of the cells on effector response. A summary of these results is shown in Fig. 4C. The histograms show that the magnitude of the downstream signaling activity (PH domain translocation) is distinctly different in cells that contain a translocation capable γ subunit, γ9, compared to others (no γ, γ3 or γ9-3). The response to 1 µM concentration of agonist in γ3 containing cells is 1.7 fold higher compared to cells expressing γ9. The response to 100 µM agonist in γ9 containing cells was 1.8 fold higher compared to cells expressing γ3. These results show that the response is significantly more sensitive to the initial low agonist concentration when the cell contains a non-translocating γ subunit. At the higher concentration of agonist only the cells expressing the translocating γ subunit show a strong additional response (Fig. 4C and D). These results confirm that cells in which receptor stimulation leads to βγ translocation are more sensitive in terms of their effector responses compared to cells in which translocation does not occur.

### FRET-based G protein sensor shows differential dissociation properties between G proteins containing translocating and non-translocating γ subunit

**Figure 5:**
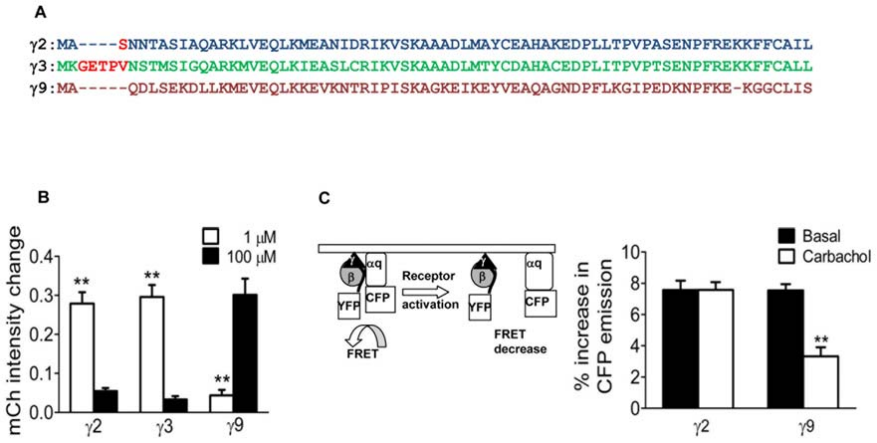
Differential effector and FRET responses of cells expressing different γ subunits. *A.* Primary structures of γ3, γ2 and γ9. Comparison shows that γ3 has five additional residues at the N terminus compared to γ9 while γ2 has only one (highlighted residues). *B.* PH-mCh translocation induced by the sequential addition of agonist from cells expressing different γ subunit types (as indicated). N = 11. Results are the means ± SEM. *p<0.01, **p<0.001. *C.* A FRET sensor based on αq-CFP and βγ-YFP. Representation of FRET sensor (left). Bars (right) show increase in CFP emission on the plasma membrane as a result of FRET loss in presence of various γ subunits at basal (black bars) and activated (white bars) state determined as in Methods and Text S1. Results are the means ± SEM; N = 9, **p<0.001.

To examine the mechanism at the basis of the effect of different γ subunits on the sensitivity of a cell to activation, we used a FRET based sensor containing αq-CFP and YFP-γ. The design of this sensor was based on a previous sensor we had developed using αo [14]. Briefly, the donor CFP is tagged to αq subunit and acceptor YFP to Gγ. In the basal state due to formation of G protein heterotrimer there is FRET from CFP to YFP leading to diminished emission from CFP. When the G protein heterotrimer is activated by an active GPCR, if there is dissociation of Gα and Gβγ then the FRET is lost leading to restoration of CFP intensity which is marked by an increase in its fluorescence emission. We observed the FRET signal in live M3-CHO cells expressing αq-CFP and YFP-γ3 or YFP-γ9. Only cells expressing comparable intensities of CFP and YFP proteins were selected for observation. FRET experiments were performed as described before [14], [19]. Essentially, FRET was determined by monitoring gain in CFP emission intensity in the plasma membrane by photobleaching YFP (acceptor photobleaching [20]) in both the basal and agonist activated states. We did not detect any FRET from αq-CFP YFP-γ3 in the basal state (data not shown) while αq-CFP YFP-γ9 did provide a clearly detectable FRET signal. To ensure that the lack of detectable FRET between αq-CFP and βYFP-γ3 is not due to the inability of these subunits to form a heterotrimer, we examined the ability of αq-CFP to support the translocation of a γ3 mutant capable of translocation [7]. M3 activation of M3-CHO cells expressing αq-CFP and the γ3 mutant resulted in its translocation from the plasma membrane to internal membranes (Fig. S1). This result indicated that αq-CFP and βYFP-γ3 are capable of forming a heterotrimer. FRET does not occur between the tagged FPs likely due to five extra residues in the N terminal domain of γ3 compared to γ9 (Fig. 5A). YFP fused to the γ subunit N terminus maybe in a non-optimal orientation with reference to CFP resulting in lack of FRET between αq-CFP and YFP-γ3 [20].

To obtain an alternate FRET sensor containing a non-translocating γ subunit we examined the amino acid sequences of γ subunits and found that the γ2 sequence has only one extra residue in the N terminal domain compared to γ9 (Fig. 5A). We first examined whether the properties of γ2 were similar to γ3 by measuring the extent of PH-mCh translocation as mentioned above. The profile for PH domain translocation with γ2 (Fig. 5B and Fig. S2) was similar to that obtained with other non-translocating γ subunits (γ3 and γ9-3 chimera). We then examined the FRET signal in the basal and agonist activated states from αq-CFP to YFP-γ2 or YFP-γ9 in M3-CHO cells (Fig. 5C). In the basal state, a FRET signal was detected as a CFP increase after YFP photobleaching with both of subunits (Fig. 5C, black bars). On receptor stimulation by 100 µM carbachol, the FRET signal decreased significantly in γ9 cells but not in cells expressing γ2 (Fig. 5C, white bars). The dissociation of βγ9 from αq and its translocation away from the plasma membrane explains the reduction in the FRET signal. In surprising contrast, in the presence of γ2 the FRET signal did not change after receptor activation suggesting that either there is no dissociation of the heterotrimer or that heterotrimer reassociation is very rapid after dissociation so that it is not detected under the conditions used here. The differences in the translocation capabilities of γ subunit types thus have strikingly different impacts on heterotrimer dissociation after receptor stimulation.

These results are consistent with our model (Fig. 2) that the translocation of the βγ complex results in slower activation (cycling) of the α subunit while in the case of heterotrimers containing the non-translocating γ subunit, significant dissociation does not occur allowing much higher levels of activation by a receptor because the G protein in the heterotrimer state is available continually.

### DAG responses in cells expressing translocating or non-translocating γ subunits are similar to IP3

**Figure 6:**
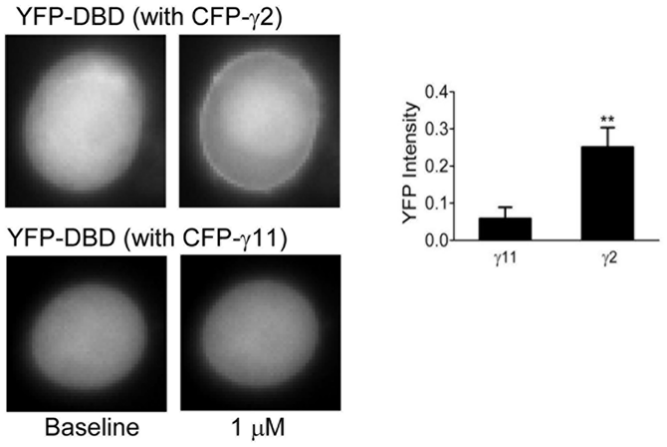
YFP-DBD translocation in the presence of various γ subunits. Representative images and bar diagrams of YFP-DBD translocation are shown in the presence of 1 µM carbachol. Values were calculated as in Fig. 1B. Images of cells expressing αq with CFP-γ2 (N = 9) or CFP-γ11 (N = 10) (as indicated) and YFP-DBD were acquired as described in Methods and Text S1. Values were calculated as ratio of M-B/B as described in Fig. 1B legend. Results are the means ± SEM. **p<0.001.

To ensure that the effects seen above by measuring IP3 levels using Ph-mCh was reflected in the generation of a different second messenger, DAG similarly in M3-CHO cells containing expressing YFP-DBD and CFP-γ11 or CFP-γ2. Cells were activated with 1 µM carbachol as above. γ11 has identical translocation properties to γ9 (Fig. S3) and was used in this case because CFP-γ11 was available, has been characterized previously and allowed us to examine whether translocation capable subunits had similar effects [6]. CFP-γ11 translocated strongly on M3 receptor activation demonstrating that the cells contained αq (Fig. S3). When M3 was activated, in cells expressing γ2, YFP-DBD translocated to the plasma membrane on activation of the receptor (Fig. 6). In contrast, cells expressing γ11 showed a weak response (Fig. 6). These results showed that regardless of the second messenger measured, cells expressing different γ subunits have differential responses.

### Endogenous γ subunit constitution influences signaling activity

We then examined whether cells that expressed an endogenous translocation proficient γ subunit showed differential response to cells not expressing any translocation proficient γ subunit. To pursue these experiments we used an αq subunit tethered to the M3 receptor to obtain equimolar concentrations of expressed receptor and αq. The N terminal of the α subunit was fused to the C terminus of the M3 receptor.

**Figure 7:**
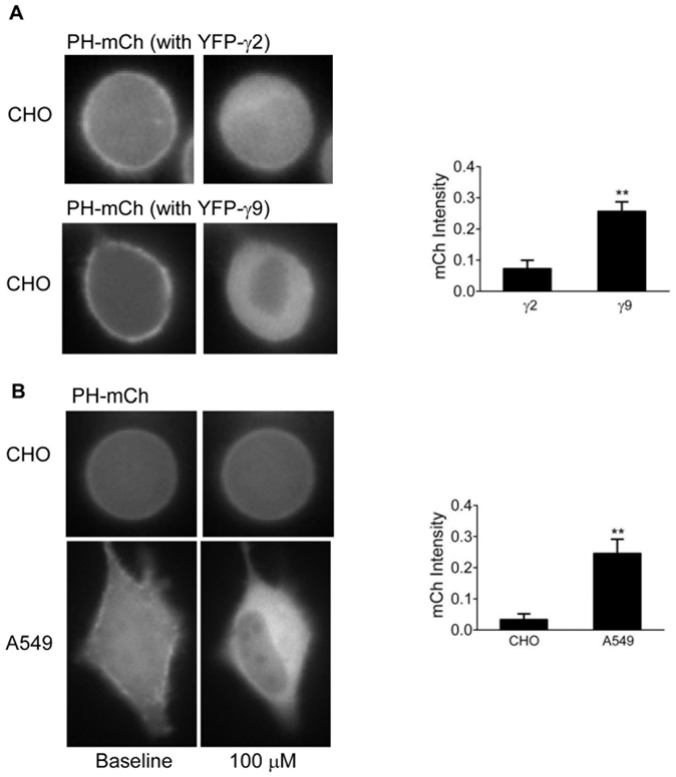
Effect of different γ subunits on M3-αq-CFP induced response. *A.* Representative images of cells expressing M3-αq-CFP with YFP-γ2 (N = 17) or YFP-γ9 (N = 9) (as indicated) and PH-mCh. Bar diagrams of PH-mCh translocation are shown in the presence of 100 µM carbachol. Values were calculated as in Fig. 1B. Results are the means ± SEM. **p<0.001. *B.* PH-mCh translocation induced by M3-αq-CFP fusion in cell lines containing endogenous non-translocating and translocating γ subunits. CHO (N = 11) and A549 (N = 13) cells expressing M3-αq-CFP, PH-mCh and no γ subunits. Values were calculated as in Fig. 1B. Results are the means ± SEM. **p<0.001.

We then confirmed the functionality of M3-αq-CFP fusion protein. The fusion protein visibly supported PH – domain translocation on activation by a M3 receptor agonist indicative of its ability to activate downstream signaling pathways like the wild type M3 and Gαq (Fig. 7).

**Figure 8:**
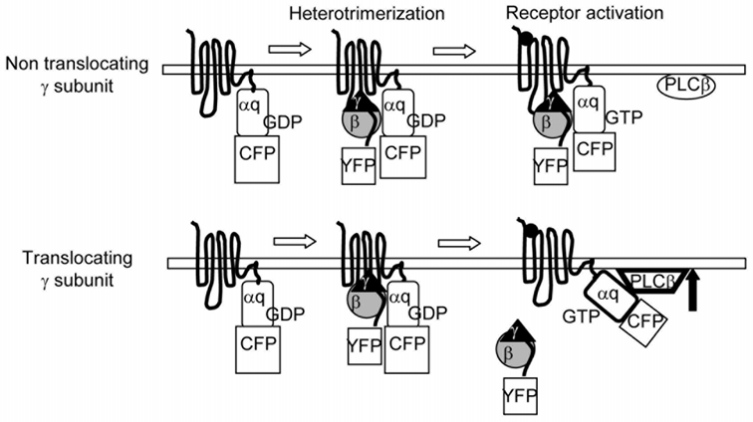
Model for the basis of differential responses to the activation of a tethered αq subunit. The tethered αq subunit when bound to a non-translocating γ subunit is unable to activate PLCβ after receptor activation because of its continued association with βγ and the inflexibility of the α subunit that is fused to the C terminus of the receptor. The effector activating surface of the αq subunit and the βγ complex do not become accessible to PLC β in this situation. In contrast, when the tethered αq subunit is bound to a γ subunit that is capable of translocation away from the plasma membrane on receptor activation, the effector activating surface of the α subunit is exposed to PLC β allowing it to activate the effector molecule.

We then evaluated PH-mCh translocation as above to determine the effector activation abilities of M3-αq-CFP in the presence of YFP-γ2 or γ9 in CHO cells. We ensured that the CFP and YFP emission intensities on the plasma membrane were similar between the γ2 and γ9 coexpressing cells (Table S1). Fig. 7A shows that in the presence of γ2, which does not effectively dissociate on receptor activation, the effector activation is very limited compared to the strong response in cells expressing the translocation capable, γ9. These results were consistent with the conformationally restricted αq not activating an effector when bound to βγ2 complex. Translocation of βγ9 allows the tethered αq to access PLC β as described in the model in Fig. 8.

These results allowed us to examine whether a cell line which contains no detectable levels of a translocating γ subunit (CHO) or one that contains a translocating γ subunit (A549) demonstrate properties that are consistent with a role for these subunits in the regulation of signaling sensitivity in a cell. We screened a variety of cell lines for the presence of endogenous translocating γ subunits by checking for the potential cotranslocation of YFP-β1 introduced into these cells. We have previously shown that YFP-β1 translocates only when bound to a translocation capable γ subunit [7]. Using this approach we identified a lung epithelial fibroblast cell line, A549 containing translocation proficient γ subunits (manuscript submitted). Consistent with the ability of YFP-β1 to cotranslocate in A549 cells, qRT-PCR showed that these cells contain significant levels of translocation proficient γ11, γ10 and γ5 subunits and only one non-translocating γ subunit, γ12 (Fig. S4). In contrast to A549 cells, YFP-β1 did not cotranslocate in CHO cells (data not shown) suggesting that they do not contain translocating γ subunits at concentrations that are likely to have an effect on downstream activity. We examined these two cells types in experiments similar to those above.

When M3-αq-CFP was activated in CHO cells with 100 µM carbachol, almost no response was detected (Fig. 7B) consistent with a non-dissociating βγ preventing the conformationally restricted αq from acting on an effector. However, in A549 cells a strong response was detected consistent with the dissociation and translocation of an endogenous translocating βγ complex allowing αq to access PLCβ (Fig. 7B). These results were consistent with the model shown in Fig. 8. They also show that the endogenous γ subunit constitution can regulate the sensitivity of a cell's response to an external stimulus and the results here are not peculiar to the introduced γ subunits.

## Discussion

We have used live cell imaging of single mammalian cells to identify mechanisms at the basis of the sensitivity of cellular responses to G protein activation by an extracellular signal. We show here that the ability of G protein γ subunit types to translocate away from the plasma membrane on receptor activation controls sensitivity of response. Cells that express a translocating γ subunit demonstrate attenuated PIP2 breakdown in response to M3 receptor stimulation compared to cells that express a non translocating γ subunit. The lowered sensitivity is not due to a specific property of a γ subunit type but due to the translocation of the βγ complex because cells expressing a translocation capable γ subunit mutated to abolish translocation showed heightened sensitivity similar to non translocating γ subunits.

We examined the basis of this differential effect of translocating and non-translocating γ subunits using a FRET sensor based on αq-CFP and YFP-γ interaction. The Gq sensor behaved similar to a Go sensor when it contained γ9, demonstrating a significant loss in FRET on receptor activation. But surprisingly with a non-translocating γ subunit, γ2, there was no loss in FRET on receptor activation. The lack of βγ2 dissociation from αq is consistent with a long standing suggestion that the G protein heterotrimer does not have to dissociate to act on effectors [21], [22] and recent reports of the inability of receptor activation to dissociate αq, αi-1, αz, αt-cone and αs-q chimera from β1γ2 [23], [24], [25], [26], [27]. This result is also consistent with our previous finding that βγ translocation is influenced by the associated α subunit type [28]. The lack of dissociation of αqβγ2 (or very brief dissociation) allows continual access to a receptor, a higher level of activation of αq and high level of PLCβ activation and PIP2 breakdown (Fig. 2). In contrast, in the case of αqβγ9, translocation of βγ results in reduction of heterotrimer available on the plasma membrane for PLCβ activation resulting in lowered PIP2 breakdown (Fig. 2).

To further examine the role of subunit dissociation and translocation on the sensitivity of the cell's response to an agonist we tethered the α subunit to the receptor so that it is conformationally constrained. Similar receptor α subunit fusions have previously been shown to be capable of functioning effectively on receptor activation [29]. PIP2 breakdown was strong in cells containing the M3-αq-CFP protein and translocating γ9 and weak in cells expressing non-translocating γ2. The results support the model in Fig. 8. Thus tethered αq is unable to access PLCβ when bound to a non dissociating βγ complex. In contrast, in the presence of the translocating βγ9 subunits, the tethered αq is able to activate PLCβ because it is accessible to PLCβ after βγ dissociation and translocation.

While mechanisms that desensitize a cell's response to external stimuli are well characterized at the level of the receptor [30], few mechanisms that act at the level of the G protein to attenuate or accentuate the response to a signal have been identified. In the Drosophila compound eye, DGqα, translocates from the rhabdomere, the membrane structure containing the phototransduction machinery, to the cell body (Golgi/ER) upon rhodopsin activation with a time course of a few minutes and reverse translocates on rhodopsin inactivation with a time course of tens of minutes [31]. This translocation of DGqα has been shown to regulate the sensitivity of the phototransduction cascade by reducing the amount of DGqα following illumination [32]. In rod photoreceptors, translocation of Gt subunits from outer to inner segment on rhodopsin activation has been shown [5], [33]. This translocation has been shown to help adapt to the light signal by attenuating the response [33], [34]. It is of interest that the G protein in the rods contains γ1 that we have previously shown to be capable of translocation when activated by a GPCR [7]. However, there are several striking distinctions between the properties noted for translocation in rod photoreceptors and the translocation of the G protein βγ complex examined here. Activated rhodopsin induces the translocation of both the α and βγ subunits of Gt unlike the translocation examined here. The translocation of Gt subunits also occurs at a much slower rate than the selective βγ translocation studied here (t1/2: αt ∼5 min and βγt ∼12 min [33] in contrast to βγ9 ∼10 sec [7]). Additionally, the reversal of G protein subunit translocation in the rods occurs much slower than its forward translocation (200 min for αt) [35] compared to βγ9 which reverses at the same rate as forward translocation [7]. Finally, there is no equivalent in rod photoreceptors to the heightened response seen in the case of non-translocating γ subunits. It is however possible that light adaptation conferred by the translocation of G protein subunits in rod photoreceptors is evolutionarily related to the attenuation of effector response seen here.

An important question is whether the results we have seen are of significance to the sensitivity of response of mammalian cells into which γ subunits have not been introduced. In CHO cells that do not contain a translocating γ subunit and in A549 cells that contain translocating γ subunits, the M3 tethered αq induced distinctly different PIP2 breakdown responses that were consistent with the responses seen in cells into which non-translocating γ2 or translocating γ9 subunits were introduced. These results showed that endogenous γ subunit type constitution of cells can regulate the sensitivity of a cell's response to an external stimulus.

Previously we have shown that a FRET based sensor made up of αo and βγ2 shows FRET loss on receptor activation [14] suggesting that non-translocating βγs dissociate from some α subunits. In contrast, the results here suggest that αq does not dissociate from βγ2 on activation. Thus two different factors appear to control the concentration of heterotrimer available to the activated receptor. The α subunit type which affects dissociation and the γ subunit type which regulates translocation and consequently dissociation. Signaling responses can thus be controlled by the α and γ subunit type constitution of a cell. Consistent with this prediction, we have previously reported that in a receptor reconstituted system, both the α subunit type and the γ subunit type affected the receptor stimulated activity of G proteins [36].

G proteins control the vast majority of signaling pathways in mammalian cells and the existence of diverse evolutionarily conserved families of G protein α and γ subunits has been known for many years [1]. But the roles that these subunit types play in the dynamic environment of living cells have not been fully explored [37]. While there has been evidence for the specificity of interaction between G protein α and γ subunits with receptors [38], [39], their roles in regulating the kinetics of signaling in mammalian cells has been poorly defined. The results here identify mechanisms that allow the existent diversity of G protein subunits in a cell to control the intensity of response to varying levels of external stimuli. Overall, these results suggest that G protein βγ complex translocation and dissociation of the heterotrimer are novel mechanisms that can control the intensity of response of a cell to receptor stimulation.

## Materials and Methods

### Expression constructs and cell culture

γ subunits were tagged with YFP or CFP as described previously [6], [7]. The chimera γ9-3 was made by substitution of the last 15 amino acids of the C terminus from γ3 in γ9 subunit. αq-GFP (from C. Berlot, Geisinger Clinic) was mutated to obtain αq-CFP and was transferred to pcDNA3.1. PH domain of PLC δ was obtained as PH-EGFP [13] (from T. Balla, NIH) and GFP was substituted with mCh (from R. Tsien, UC San Diego). YFP-DBD was obtained from A. Newton, UC San Diego [11].

CHO cells stably expressing the M3 muscarinic receptor (M3-CHO) have been described previously [14]. CHO cells were grown in CHO IIIa medium (Invitrogen) containing 10% dialyzed fetal bovine serum (Atlanta Biologicals), methotrexate, penicillin, streptomycin, and glutamine. A549 cells were grown in Ham's F12 (Mediatech) with 10% dialyzed fetal bovine serum, penicillin and streptomycin. All the transfections were performed using Lipofectamine 2000 (Invitrogen) as described previously [19] (additional details are in Text S1).

### Live cell imaging

Briefly, transfected cells cultured on glass coverslips were imaged in a chamber with a 25 µl volume using a Zeiss Axioskop microscope with a 63x objective (1.4 NA), 100 W mercury arc lamp and Hamamatsu CCD Orca-ER camera. There was programmed delivery of solutions at 0.5 ml/min flow rate using an automated delivery system (Automate Scientific). For FRET experiments, cells with equal expression levels of CFP and YFP were selected and experiments were performed as previously described [14]. Chromatic filters and other details are in Text S1.

## Supporting Information

Figure S1 M3-CHO cells were co-transfected with αq-CFP and YFP-γ3 C-terminal mutant. M3 activation induces translocation of the γ3 mutant. Images from transfected cells were acquired with 10 sec intervals. Cells were exposed to 100 µM carbachol (agonist) followed by a wash with buffer at the indicated time points (as shown by arrows in plot). YFP emission intensity changes over time in Golgi (white arrow) were plotted.
(0.02 MB PDF)

Figure S2 M3-CHO cells were co-transfected with αq-CFP, YFP-γ2 and PH-mCherry. Images from transfected cells were acquired with 10 sec interval. Cells were exposed sequentially to 1 µM and 100 µM carbachol (agonist) followed by a wash with buffer at the indicated time points (as shown by arrows in plot). YFP changes over time in Golgi and mCherry emission intensity in cytosol (as indicated by black circles) were plotted.
(0.03 MB PDF)

Figure S3 M3-CHO cells were co-transfected with αq, CFP-γ11 and YFP-DBD. Images from transfected cells were acquired with 10 sec interval. Cells were exposed to 1 µM carbachol (agonist) followed by a wash with buffer. As shown in these images, CFP-γ11 translocates with the same efficiency as YFP-γ9. Images of YFP-DBD are shown in main text Fig. 6 (same cell).
(0.01 MB PDF)

Figure S4 Real time PCR analysis of the relative expression levels of the G protein gamma subunit family
(0.11 MB PDF)

Table S1 CFP emission intensities are not correlated with cell responses
(0.02 MB PDF)

Text S1 Materials and Methods
(0.04 MB PDF)

Text S2 Effect of G protein βγ on PLCβ activation
(0.01 MB PDF)
