# Supplementary figures and images for: G Protein Subunit Dissociation and Translocation Regulate Cellular Response to Receptor Stimulation
Source: PLoS One. 2009 Nov 11;4(11):e7797. doi: 10.1371/journal.pone.0007797 (PMC2777387; doi:10.1371/journal.pone.0007797)

**Fig. S1**

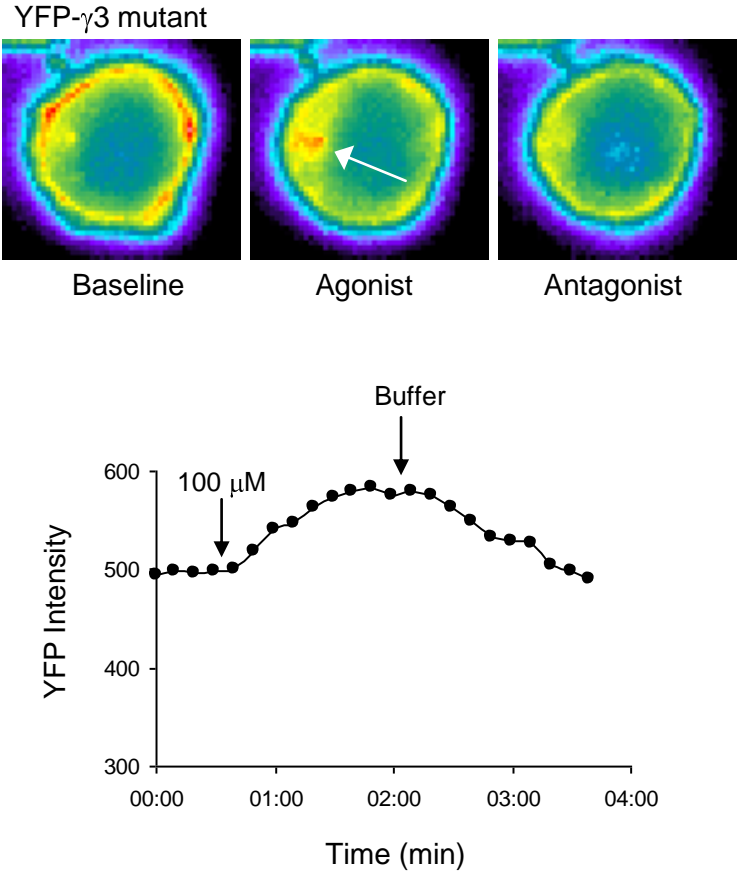

Supplement: Figure S1 — M3-CHO cells were co-transfected with αq-CFP and YFP-γ3 C-terminal mutant. M3 activation induces translocation of the γ3 mutant. Images from transfected cells were acquired with 10 sec intervals. Cells were exposed to 100 µM carbachol (agonist) followed by a wash with buffer at the indicated time points (as shown by arrows in plot). YFP emission intensity changes over time in Golgi (white arrow) were plotted. (0.02 MB PDF) [file pone.0007797.s001.pdf]

**Fig. S2**

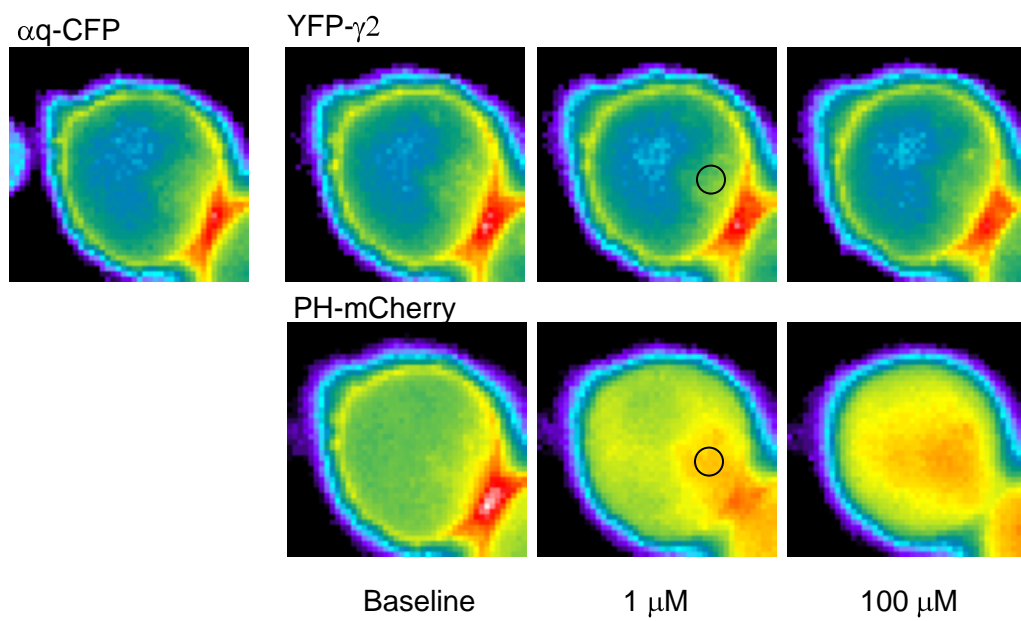

Supplement: Figure S2 — M3-CHO cells were co-transfected with αq-CFP, YFP-γ2 and PH-mCherry. Images from transfected cells were acquired with 10 sec interval. Cells were exposed sequentially to 1 µM and 100 µM carbachol (agonist) followed by a wash with buffer at the indicated time points (as shown by arrows in plot). YFP changes over time in Golgi and mCherry emission intensity in cytosol (as indicated by black circles) were plotted. (0.03 MB PDF) [file pone.0007797.s002.pdf]

**Fig. S3**

CFP- $\gamma$ 11

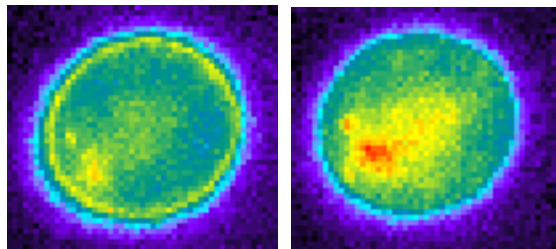

Baseline

1  $\mu$ M

Supplement: Figure S3 — M3-CHO cells were co-transfected with αq, CFP-γ11 and YFP-DBD. Images from transfected cells were acquired with 10 sec interval. Cells were exposed to 1 µM carbachol (agonist) followed by a wash with buffer. As shown in these images, CFP-γ11 translocates with the same efficiency as YFP-γ9. Images of YFP-DBD are shown in main text Fig. 6 (same cell). (0.01 MB PDF) [file pone.0007797.s003.pdf]
