# Supplementary material for: G Protein Subunit Dissociation and Translocation Regulate Cellular Response to Receptor Stimulation
Source: PLoS One. 2009 Nov 11;4(11):e7797. doi: 10.1371/journal.pone.0007797 (PMC2777387; doi:10.1371/journal.pone.0007797)
Supplement: Figure S4 — Real time PCR analysis of the relative expression levels of the G protein gamma subunit family (0.11 MB PDF) [file pone.0007797.s004.pdf]

Fig. S4

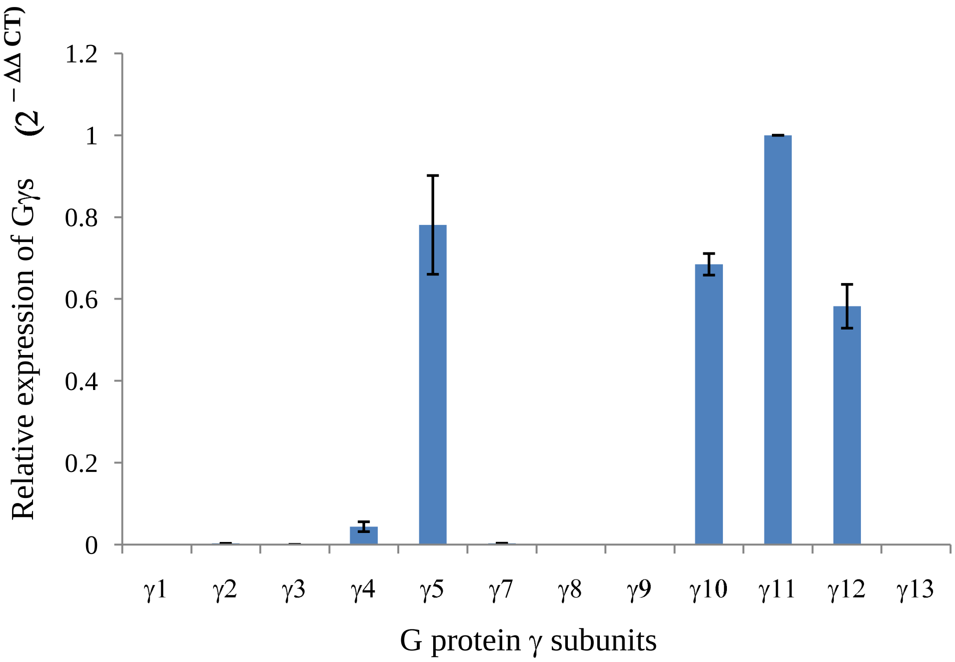

Real time PCR analysis of the relative expression levels of the G protein γ subunit family. γ11 is the preponderantly expressed γ subunit in A549. Relative expression levels of subunits ($2^{-\Delta\Delta C_T}$) were determined with reference to that of γ11 after normalizing the expression of individual subunits to that of β-actin. Bars are ± SEM (n=3). In a positive control using cDNAs for the γ subunit types, all primers amplified expected products.

Methods: Total RNA was extracted from A549 cells using RNeasy Plus Mini Kit (Qiagen). For cDNA synthesis, 1 μg RNA was used in a 20 μl Reverse Transcriptase reaction (ThermoScript RT-PCR system from Invitrogen). Real time PCR was performed in a 7500 Real Time PCR System (Applied Biosystems). For each reaction, 10 ng of the cDNA was used in a 20 μl reaction containing 10 μl of the Power SYBR Green PCR Master Mix (Applied Biosystems). Each reaction was performed in triplicate.

The following primers were used in the qRT-PCR analysis –

γ1, GAAAGAAGTGACACTGGAAAG and CTCTGGGATGCCCTTTAC;

γ2, GCTGGTAGAGCAGCTTAAGATG and GGTCTTCCTTGGCATGTGC;

γ3, CAGCAGCAGACCTGATGAC and GAACTTCTTCTCCCGGAAGG;

γ4, CTGTGGAGCAGCTAAAGATG and GATGAGAGGATCTTCCCG;

γ5, GAAGAAAGTGGTTCAACAGC and GCAGACAGAACTGTTTCAAGTC;

γ7, GCATAGAA GCCGGGATTGAG and CAGGGACTCCGACCAGCAG;

γ8, GCAAGACGGTGGAACAGC and CATCTTTGGCATGCGTCTC;

γ9 GTTGAAGATGGAGGTGGAGC and CTCCACGTAC TCCTTGATTTCC;

γ10, CAGCGCTTGGTAGAGCAG and CATCCTTGCAGGCATTCTG;

γ11, GAAGTGAAGTTGCAGAGACAAC and CTGGAATTCCCTTTACTAGAGG;

γ12, GTGCA GCAGTTAAGATTAGAAGCC and CTCCTGGCATGTTCCTCAC;

γ13, CAAGTACCAGCT GGCCTTC and GTTCAGGAAGGGGTCCTTG.
