# Supplementary material for: G Protein Subunit Dissociation and Translocation Regulate Cellular Response to Receptor Stimulation
Source: PLoS One. 2009 Nov 11;4(11):e7797. doi: 10.1371/journal.pone.0007797 (PMC2777387; doi:10.1371/journal.pone.0007797)
Supplement: Table S1 — CFP emission intensities are not correlated with cell responses (0.02 MB PDF) [file pone.0007797.s005.pdf]

Table S1

CFP emission intensities are not correlated with cell responses.

The M3-αq-CFP was localized to regions corresponding to the plasma membrane and the cell interior. The intensity of CFP emission was measured from the plasma membrane region. We selected mostly cells that had similar levels of CFP emission intensity which is an indicator of the level of expression of M3-αq. The YFP-γ2 and YFP-γ9 emission intensities were also similar. So the differential responses of γ2 and γ9 cells as well as CHO and A549 cells (Fig. 7) to the activation of M3-αq are not due to differences in the expression levels of the M3-αq-CFP fusion protein. The table below shows that CFP emission intensities of individual cells do not correlate with their responses to receptor activation. Expression levels of M3-αq-CFP in CHO cells are higher in the absence of a subunit because there is no competition from another vector.

γ2		γ9		CHO		A549	
CFP	Response	CFP	Response	CFP	Response	CFP	Response
425	0	293	0.27	670	0	498	0.35
270	0.07	308	0.22	578	0	483	0.17
368	0	287	0.22	500	0.13	480	0.2
400	0	296	0.16	690	0	600	0.44
283	0	336	0.35	510	0	600	0.41
390	0	390	0.27	640	0	500	0.2
385	0.27	416	0.17	760	0.08	380	0.4
365	0.1	432	0.41	900	0	340	0.33
