# Supplementary material for: G Protein Subunit Dissociation and Translocation Regulate Cellular Response to Receptor Stimulation
Source: PLoS One. 2009 Nov 11;4(11):e7797. doi: 10.1371/journal.pone.0007797 (PMC2777387; doi:10.1371/journal.pone.0007797)
Supplement: Text S1 — Materials and Methods (0.04 MB PDF) [file pone.0007797.s006.pdf]

Text S1 Materials and Methods.

Constructs and cell culture. All subunits (γ2, γ3, γ3 C-terminal translocating mutant, γ9 and γ11) tagged with yellow fluorescent protein (YFP) or cyan FP (CFP) constructs have been described previously [1,2]. The chimera γ9-3 was made by substitution of the last 15 amino acids of the C terminus from γ3 in γ9 subunit. PH domain of PLC was obtained by PH-EGFP (from T. Balla [3]) and then the GFP was substituted with mCherry fluorescent protein (from R. Tsien) [4]. YFP-DBD was obtained from A. Newton [5]. The αq-GFP in pcDNA1 (from C. Berlot, [6]) was used as a template in a Quik Change Multi Site Directed Mutagenesis reaction (Stratagene) and the GFP within the alpha-q was mutated to CFP. The entire αq-CFP cDNA was then transferred to the HindII and NotI sites of pcDNA3.1.

CHO cells stably expressing the M3 muscarinic receptor (M3-CHO) have been described previously [7]. CHO cells were grown in CHO IIIa medium (Invitrogen) containing dialyzed fetal bovine serum (Atlanta Biologicals), methotrexate, penicillin, streptomycin, and glutamine. A549 cells were grown in Ham's F12 (Mediatech) with dialyzed fetal bovine serum, penicillin and streptomycin. All the transfections were performed using Lipofectamine 2000 (Invitrogen) as described previously [8].

Live Cell Imaging. Live cell imaging experiments were performed as follows. Cells were cultured on glass coverslips and transiently transfected with different cDNAs (details are in figure legend and text) using Lipofectamine 2000. After 16-24 h post-transfection, cells were washed with Hanks' buffered saline solution supplemented with 10 mM HEPES, pH 7.4, and mounted on an imaging chamber with an internal volume of 25 μl (RC-30 chamber, Warner Instruments). A fluid delivery system including a

programmable valve controller and Teflon valves (Automate Scientific) was used to deliver buffer and agonist through the chamber at a rate of 0.5 - 0.6 ml/min with a regulated flow controller. The cells were visualized with a Zeiss Axioskop fluorescent microscope using a 63x oil immersion objective (1.4 NA) and 100-watt mercury arc lamp with a Hamamatsu CCD Orca-ER camera. The shutter and emission and excitation filter wheels were controlled by a Sutter Lambda 10-2 optical filter changer (Sutter Instrument Co.) run by MetaMorph 6.3.7 (Molecular Devices) software. The filter and beam splitter combinations (Chroma Technology) were as follows; for CFP, D436/10 excitation, D470/30 emission; for YFP, D500/20 excitation, D535/30 emission; for mCherry or DsRed D580/20 excitation, D630/60 emission, a polychroic beam splitter (Chroma 86002BS) and 10% neutral density filters. In FRET experiments, the FRET signal was determined by monitoring gain in CFP emission intensity in plasma membrane by photobleaching of YFP (acceptor photobleaching) [7]. Cells with equal expression levels of CFP and YFP were selected and experiments were performed as previously described [7]. Images for CFP were acquired before and after YFP photobleaching and intensity levels were compared and normalized to the prebleaching intensity. The emission intensity was corrected for CFP bleaching by determining it in cells expressing αq-CFP alone. In agonist treated cells, the cells were stimulated with 100 M carbachol before YFP photobleaching.

Although the citrine version of YFP which is less prone to bleaching was used, strong bleaching was observed in experiments where the images were captured in the streaming mode with 1 sec exposure time. In this mode images of cells were captured continuously with no interval time during acquisition. This bleaching does not affect the interpretation

of results shown. In other experiments, the interval time for acquisition was increased to 10 sec to minimize bleaching.

References

1. Saini DK, Kalyanaraman V, Chisari M, Gautam N (2007) A family of G protein betagamma subunits translocate reversibly from the plasma membrane to endomembranes on receptor activation. J Biol Chem 282: 24099-24108.
2. Akgoz M, Kalyanaraman V, Gautam N (2004) Receptor-mediated reversible translocation of the G protein betagamma complex from the plasma membrane to the Golgi complex. J Biol Chem 279: 51541-51544.
3. Varnai P, Balla T (1998) Visualization of phosphoinositides that bind pleckstrin homology domains: calcium- and agonist-induced dynamic changes and relationship to myo-[3H]inositol-labeled phosphoinositide pools. J Cell Biol 143: 501-510.
4. Zhang J, Campbell RE, Ting AY, Tsien RY (2002) Creating new fluorescent probes for cell biology. Nat Rev Mol Cell Biol 3: 906-918.
5. Gallegos LL, Kunkel MT, Newton AC (2006) Targeting protein kinase C activity reporter to discrete intracellular regions reveals spatiotemporal differences in agonist-dependent signaling. J Biol Chem 281: 30947-30956.
6. Hughes TE, Zhang H, Logothetis DE, Berlot CH (2001) Visualization of a functional Galpha q-green fluorescent protein fusion in living cells. Association with the plasma membrane is disrupted by mutational activation and by elimination of palmitoylation sites, but not be activation mediated by receptors or AlF4. J Biol Chem 276: 4227-4235.
7. Azpiazu I, Gautam N (2004) A Fluorescence Resonance Energy Transfer-based Sensor Indicates that Receptor Access to a G Protein Is Unrestricted in a Living Mammalian Cell. J Biol Chem 279: 27709-27718.
8. Chisari M, Saini DK, Kalyanaraman V, Gautam N (2007) Shuttling of G protein subunits between the plasma membrane and intracellular membranes. J Biol Chem 282: 24092-24098.
