# Supplementary material for: G Protein Subunit Dissociation and Translocation Regulate Cellular Response to Receptor Stimulation
Source: PLoS One. 2009 Nov 11;4(11):e7797. doi: 10.1371/journal.pone.0007797 (PMC2777387; doi:10.1371/journal.pone.0007797)
Supplement: Text S2 — Effect of G protein βγ on PLCβ activation (0.01 MB PDF) [file pone.0007797.s007.pdf]

**Text S2**

Effect of G protein βγ on PLCβ activation. CHO cells stably expressing M2 muscarinic receptor were transiently transfected with αo, β1, YFP-γ9 subunits and PH-mCh sensor. The cells were imaged as described in the materials and methods section. Briefly, they are mounted on imaging chamber and sequentially exposed to 100 μM of M2 receptor agonist (carbachol) followed by 100 μM of antagonist (atropine). Images for YFP-γ11 and PH-mCh were captured at every 10 sec interval. Translocation of YFP-γ11 in response to M2 receptor activation was observed indicating that receptor activation status. The translocating βγ reverse translocated on plasma membrane on deactivation of the receptor. On the other hand no change in localization of PH-mCh was observed indicating towards failure of Gβγ to activate PLCβ which leads to PIP2 hydrolysis. Substitution of Gγ11 with other gamma subunits (γ2 or γ3) has no impact on the observations.

To ascertain that the cells are not mutated for PLCβ activity, transient introduction of a Gαq coupled receptor, M3 induced significant translocation of PH-mCh indicating that the cells used in the study were completely functionally proficient. These observations clearly indicated that no PLCβ activation through Gβγ in living cells is detected.
